# Impact of Nutrition Education on the Nutrition Capacity of Caregivers and Nutrition Outcomes of Indigenous Mbororo Children in the West Region of Cameroon: Protocol for a Cluster Randomized Controlled Trial

**DOI:** 10.2196/23115

**Published:** 2021-05-20

**Authors:** Florence Titu Manjong, Vincent Siysi Verla, Thomas Obinchemti Egbe, Dickson Shey Nsagha

**Affiliations:** 1 Department of Public Health and Hygiene Faculty of Health Sciences University of Buea Buea Cameroon; 2 Department of Pharmacy Technology St Louis University Institute of Health and Biomedical Sciences Bamenda Cameroon; 3 Department of Internal Medicine and Pediatrics Faculty of Health Sciences University of Buea Buea Cameroon; 4 Department of Obstetrics and Gynecology Faculty of Health Sciences University of Buea Buea Cameroon

**Keywords:** nutrition education, caregivers, nutrition outcomes, indigenous children

## Abstract

**Background:**

Inadequate diets and life-threatening infections have profound adverse implications for child growth, development, and survival, particularly among indigenous peoples. Evidence of the effectiveness of community-based nutrition education interventions in improving child feeding and nutrition outcomes among indigenous Mbororo population in Cameroon is scarce.

**Objective:**

This study aims to investigate the impact of culturally tailored community-based nutrition education intervention on caregivers’ knowledge, attitude, and practice regarding complementary feeding and on nutrition outcomes of indigenous Mbororo children (aged 3-59 months) in the Foumban and Galim health districts of the West Region of Cameroon.

**Methods:**

A two-arm cluster randomized controlled trial will be conducted in the Foumban Health District and Galim Health District. The intervention and control arms will each comprise 5 clusters with 121 child–caregiver pairs. Participants in the intervention arm will be organized into 5 caregivers’ peer-support platforms. A total of 12 educational sessions will be assigned to the intervention group by trained female Mbororo nutrition volunteers (n=6) and community health workers (n=6). The control arm will receive routine facility-based nutrition education. Data will be collected at 3-month and 6-month follow-up. Both descriptive statistics and multivariate logistic models will be used to estimate the effect of culturally tailored community-based nutrition education intervention (independent variable) on outcome variables (caregivers’ knowledge, attitude, and practice), child growth (weight, height/length, weight for age), and morbidity status (diarrhea, cough, and fever) between both arms. Data assessors will be blinded to the group allocation. Ethical approval (reference no. 2019/1002-07/UB/SG/IRB/FHS) was obtained from the Faculty of Health Sciences Institutional Review Board at the University of Buea.

**Results:**

Baseline data were collected in September 2019. In February 2020, 10 Mbororo communities (clusters) with 242 child–caregiver pairs were selected and allocated to the experimental and control arm in a 1:1 ratio. Community nutrition volunteers (n=6) and community health workers (n=6) were selected and trained. Data collection and analysis are ongoing, and results are not available for this manuscript.

**Conclusions:**

The findings of this study will provide evidence on the impact of culturally tailored and health belief model–based nutrition education on behavior change as a complementary strategy for strengthening health facility–based approaches in the reduction of malnutrition burden among the study population

**International Registered Report Identifier (IRRID):**

DERR1-10.2196/23115

## Introduction

Every year, 5.6 million children die before their fifth birthday, with 80% of these deaths occurring in sub-Saharan Africa and Asia [1]. Malnutrition accounts for more than 45% of all child deaths globally [2]. Thus, achieving the Sustainable Development Goal Target 2.2 [3] of ending all forms of malnutrition will significantly contribute to the reduction of child mortality to 25 deaths per 1000 live births in every country by 2030 [3]. Effective malnutrition reduction strategies hinge largely on reducing key underlying factors, particularly among those groups identified as high-risk according to evidence-based approaches [4].

Poor diets and life-threatening infections during childhood, such as diarrhea and pneumonia, are the immediate and major causes of childhood undernutrition in developing countries [1,5]. Low maternal socioeconomic status, inadequate nutrition capacity, limited access to health care, and poverty are important contributing factors [1]. In particular, inadequate maternal knowledge regarding exclusive breastfeeding and complementary feeding practices has been implicated in childhood undernutrition [5,6]. In Cameroon, more than half of children aged 6-23 months do not receive adequate complementary feeding, and only 33% of these children receive the minimum dietary diversity [7]. Improving mothers’/caregivers’ child-feeding behavior is thus an important target and has the potential to improve child nutritional status and survival.

Community‐based programs are unique platforms for the delivery of nutrition interventions [8]. Pooled analyses show that home-based and community-based nutrition education interventions for mothers improve the nutritional status of children younger than 5 years in developing countries [9,10]. Similarly, hygiene counseling components of interventions significantly decrease diarrhea episodes and dysentery among children younger than 5 years [11]. Intervention delivery strategies, such as home visiting, conducting group meetings of caregivers and community leaders, providing education regularly, and the use of cooking demonstrations, have been shown to produce positive outcomes [9].

In Cameroon, the nutrition education for mothers is predominantly facility based. Within the context of a weak health care system, characterized by low coverage, underresourcing, and workforce shortage [12], community-based strategies are needed to complement and strengthen facility-based approaches. Further, facility-based education often fails to adequately address context-specific barriers to behavior change. Moreover, the potential for community-based nutrition education targeting the Mbororo population in Cameroon has not been adequately explored. As an ethnic minority indigenous people [13], the Mbororo reside predominantly in hard-to-reach rural settings where child malnutrition rates are disproportionately higher [7]. It is worth nothing that indigenous children experience higher nutrition-related problems than their nonindigenous counterparts worldwide [14,15]. It is against this background that this study is designed to evaluate the effect of a nutrition education intervention on caregivers’ nutrition-related knowledge, attitude, and practice (KAP) and nutrition outcomes of indigenous Mbororo children younger than 5 years in the study area. The study design is informed by the health belief model and contextual realities of the target community obtained from the formative study. The study is innovative and culturally tailored to ensure acceptability and sustainability. The findings will contribute to the growing body of evidence on community-based nutrition education for behavior change as a complementary strategy for strengthening health care systems and achieving overall child health–related goals in Cameroon.

## Methods

### Trial Registration Status

The protocol was submitted to the Wealth Health Organization (WHO) Pan Africa Clinical Trial Registry in South Africa for review. Feedback is still being awaited. The last correspondence was on July 4, 2020.

### Trial Design

**Figure 1:**
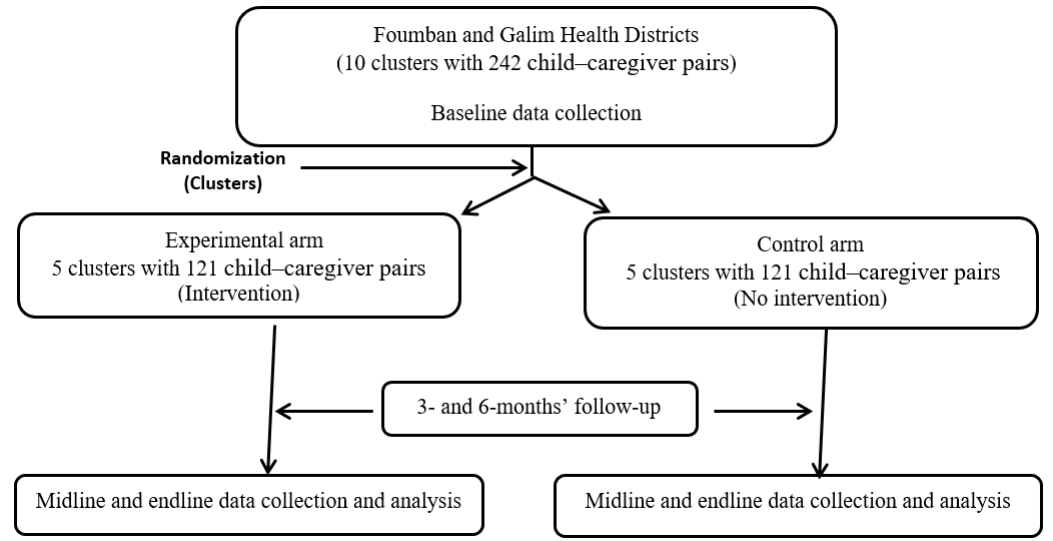
Flow chart showing cluster randomization and follow-up for the nutrition, water, sanitation, and hygiene (Nu-Wash) model.

As presented in Figure 1, this is a 2-arm parallel intervention study designed as a cluster randomized controlled trial. The randomization units will be clusters to prevent contamination between the experimental and control arms. A computer-generated list of 10 randomly selected clusters will be produced and placed in sealed, opaque envelopes.

The clusters will be allocated in a 1:1 ratio in both arms. The study will be single blinded, as only the data assessors (data collectors and analysts) will be blinded to the group allocations. Within each cluster, children and caregivers meeting the inclusion criteria will be recruited for the study. Informed consent and assent will be obtained from caregivers before the intervention. Caregivers in the experimental arm will receive nutritional education under the nutrition, water, sanitation, and hygiene (Nu-WASH) model, and caregivers in the control arm will be exposed to routine nutrition education offered by health staff in the study area. Data will be collected at 3-month and 6-month follow-up.

### Trial Setting

The study will be conducted in the West Region of Cameron with an estimated population of 1,785,285 inhabitants and a surface area of 13,960 km^2^ [16]. The population is largely rural and relies on agro- and commercial businesses for livelihoods and income. The region is host to several cultural groupings, including the indigenous Mbororo peoples, who reside in larger communities in the Bangouraim, Bangante, Foumban, Foumbot, Kouoptamo, Galim, and Mbouda health districts of the region. Foumban Health District and Galim Health District were randomly selected for the study.

### Study Participants: Inclusion and Exclusion Criteria

The study will comprise Mbororo children and their primary caregivers selected from 10 participating Mbororo communities in the Foumban Health District and Galim Health District. Participants enrolled in the study will include Mbororo households with child–caregiver pairs who participated at baseline data collection and who meet the following inclusion criteria: the children should be between 3 and 59 months of age, while their female primary caregivers should have 6 months minimum residence status, no intention to leave before the end of the study, and have provided their verbal or written informed consent and assent to participate in the study. Mbororo households without children younger than 5 years, those who did not participate at baseline data collection, those without permanent residence, children and caregivers who become seriously sick, and caregivers who refuse to participate in the study will be excluded.

### Sample Size Determination

The following formula for comparing 2 groups [17] was used for the calculation of the minimum sample size (n):


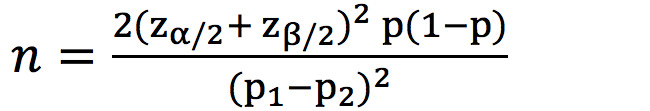


 where *z*_α/2_ = *z*_0.15/2_ = 1.44 (from *z* score table), Zβ/2 = z_0.15/2_ = 0.75 (from *z* score table at 75%), P_1_= 0.32, P_2_ = 0.22, P_1_–P_2_ = pooled prevalence (prevalence in intervention group [P1] + prevalence in control group [P2]/2):


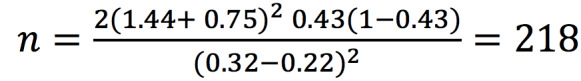


Considering the 10% nonresponse rate, the sample size (n) was adjusted to 242.

### Sampling Strategy for the Trial

**Figure 2:**
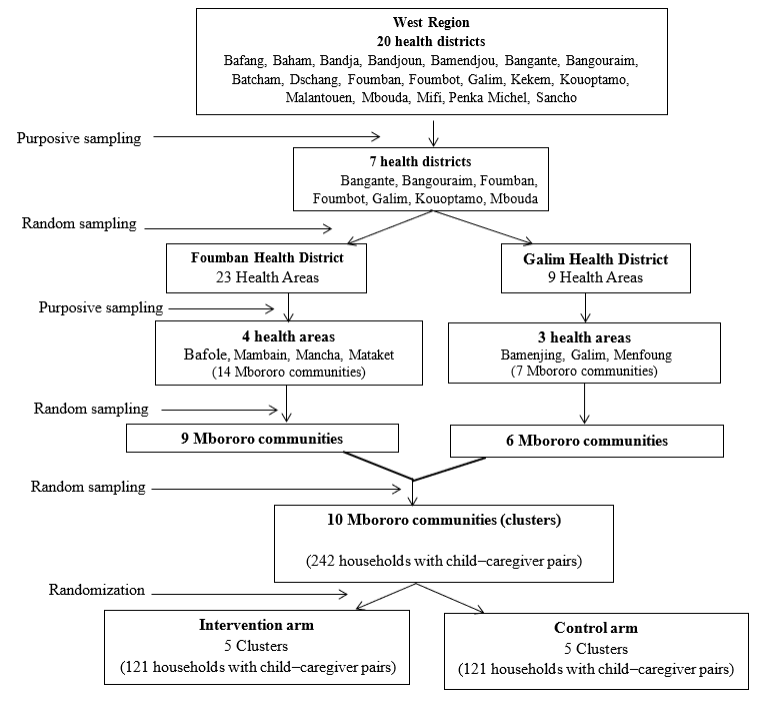
Flow chart of multistage sampling strategy for the intervention.

A multistage sampling approach, using probability and nonprobability sampling methods, was used to select the study sites and population as illustrated in Figure 2. In the first stage, Galim Health District and Foumban Health District were selected randomly by lottery method from the 7 health districts with the highest Mbororo populations. In the second stage, 7 health areas were purposively selected, and a list of Mbororo communities (n=23) was established.

### Intervention

Our Nu-WASH model comprises nutrition, and water, sanitation, and hygiene components. The nutrition component includes the following: complementary feeding, which involves the nutrition needs for young children; benefits of continuous breastfeeding for up to 24 months of child age; nutritional value of locally available plant-based and animal-based food items; dietary diversity (nutrient-rich homemade recipes); benefits and challenges of adequate complementary feeding practices; and susceptibility to, and severity of, child undernutrition. The WASH component, aimed at improving caregivers’ WASH knowledge and practices, will include the following: WASH-related childhood diseases, including causes, susceptibility, and severity; water quality, including point-of-use household drinking water treatment (boiling and filtration), and safe storage; environmental sanitation, including safe collection of child feces; personal hygiene, including proper hand washing with soap before food preparation, before child feeding, after using the toilet, after disposing of garbage, and after cleaning the baby’s feces; food hygiene (preparation, covering, serving, and heating); and the perceived barriers and benefits of food hygiene.

### Intervention Development

**Figure 3:**
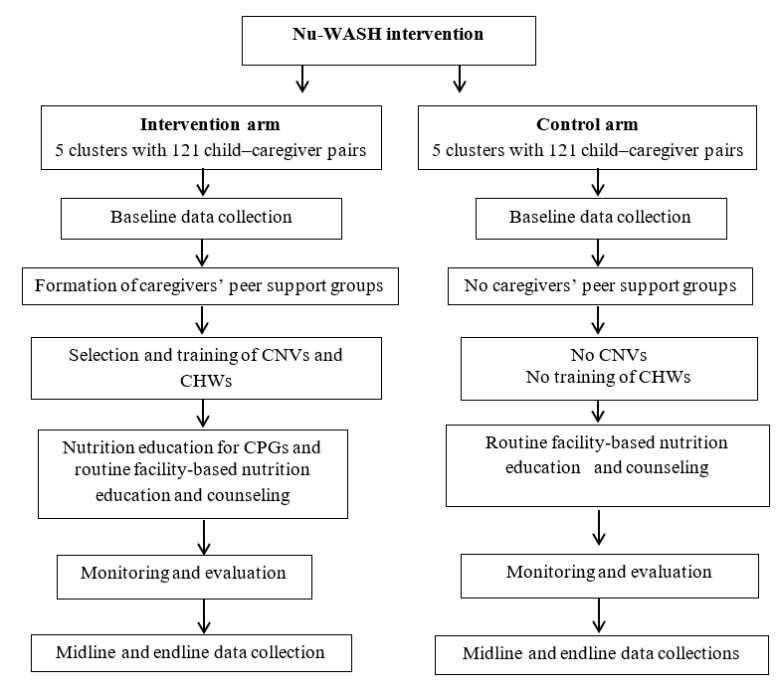
Summary of treatment given in the intervention and control arms. CHW: community health workers; CNV: community nutrition volunteers; Nu-WASH; nutrition, water, sanitation, and hygiene.

To improve on the abilities of caregivers to prevent child malnutrition, Nu-WASH will be implemented and evaluated. The target for behavior change will be caregivers assigned to the intervention arm. As illustrated in Figure 3, the intervention will be delivered through 6 logical steps: baseline data collection; the formation of caregivers’ peer support groups (CPGs); the selection and training of female community nutrition volunteers (CNVs) and community health workers (CHWs); health education for caregivers through CPGs; monitoring and evaluation; and midline and postintervention data collection and analysis.

#### Step 1: Baseline Data Collection

A formative study was conducted from August 2019 to September in 2019 to understand the local contextual factors and to inform the experimental design. A total of 10 Mbororo communities, comprising 242 mothers/caregivers and their children (aged 3–59 months), were selected for the intervention study. Baseline data on the household, caregivers, and child variables; caregivers’ nutrition-related KAP; and child anthropometric and morbidity status data were collected.

#### Step 2: Formation of CPGs

Caregivers in the intervention arm (n=121) were organized into 5 peer-support groups of 20 to 30 members. Each group per cluster will serve as the platform for group sessions and for interactions to enhance learning.

#### Step 3: Selection and Training of the CNVs and CHWs

Each participating community designated 1 Mbororo female (15 years and older) for training as a CNV. Selected CNVs and existing CHWs in the health areas were trained to lead the nutrition education and act as resource persons for nutrition information within their communities.

#### Step 4: Nutrition Education for CPGs

The CPGs in the intervention arm will be exposed to Nu-WASH and routine nutrition education offered during visits to the health care facilities in the study area. Two facilitators (1 CNV and 1 CHW) will be assigned to each CPG of the intervention arm. The intervention delivery strategy will involve group sessions and individual counseling. Each CPG will be exposed to 12 sessions: 1 session every fortnight for 6 months, each lasting for 90 to 120 minutes. A participatory and interactive delivery strategy will include lectures, discussions, question-and-answer sessions, and demonstrations. Participatory cooking demonstrations using selected combinations of food items from 7 food groups will be aimed at improving self-efficacy of preparing nutrient-rich diets. Posters showing various food groups with educational messages on nutrition (cues to action) will be provided to reinforce behavior change. Caregivers facing challenges will be given personalized counseling sessions by the CNVs and CHWs. Nutrition and WASH-related perceived barriers and benefits will be explored and discussed during the training. The control group will receive routine health facility–based nutrition and counseling offered by health care providers in the study area.

#### Step 5: Monitoring and Evaluation

CNVs and CHWs will visit households once monthly to monitor compliance and encourage proper child-feeding practices, treatment, and safe storage of drinking water. Data relevant to the study will be collected during each visit with the use of structured observation forms.

#### Step 6: Midline and Endline Data Collection and Analysis

Data will be collected from both the intervention and control arm at 3-months and 6-month follow-up with the same data collection tools, methods, and procedures as those used at baseline.

### Study Objectives

#### Primary Objective

The primary objective will be to investigate the impact of a Nu-WASH versus facility-based nutrition education on caregivers’ KAP regarding complementary feeding at baseline, 3-month follow-up, and 6-month follow-up. In the absence of a gold standard test in the measurement of KAP, composite measures will be used in this study. A composite knowledge score will be built on 4 measures based on 4 self-report questionnaire items. Knowledge will first be estimated by each measure and classified into 2 categories as adequate and inadequate before the results of the 4 measures are finally combined into the composite knowledge score. Similarly, composite scores for attitude and practice will comprise 10 and 7 measures, respectively, as derived from corresponding numbers of self-report questionnaire items.

#### Secondary Objective

The secondary objective will be to compare nutrition outcomes of Mbororo children (aged 3-59 months) at baseline, 3-month follow-up, and 6-month follow-up in the control and experimental groups. Nutrition outcomes will be defined as child growth measured as length/height, weight, and weight-for-age at baseline and endline; and child morbidity status will be measured as self-reported incidence of diarrhea, fever, and cough at 2 weeks preceding baseline, and at 3-month and 6-month data collection.

### Outcome Measurements

#### Primary Outcomes

The primary outcomes will be caregivers’ nutrition-related knowledge, and attitudes and practices of caregivers as measured using self-reported items as shown in Table 1.

#### Secondary Outcomes

As shown in Table 1, the secondary outcomes will comprise child growth (height/length, weight, and weight-for-height), which will be measured at 6-month follow-up, and child morbidity status (diarrhea, fever, and cough 2 weeks preceding baseline), which will be self-reported.

**Table 1:** Primary and secondary outcome variables among study participants at baseline, 3 months, and 6 months.

Outcome measures					Scale			Type			Measure			Analysis method
**Primary Outcomes**														
	Caregivers’ knowledge			Ratio			Continuous			Change in knowledge scores			*t* test	
	Caregivers’ attitudes			Ratio			Continuous			Change in attitudes scores			*t* test	
	Caregivers’ practices			Ratio			Continuous			Change in practices scores			*t* test	
**Secondary Outcomes**														
	**Child growth at 3 and 6 months**													
		Weight	Ratio			Continuous			Change in weight			*t* test		
		Height/Length	Ratio			Continuous			Change in height/length			*t* test		
		Weight-for-height *z* score	Ratio			Continuous			Change in weight-for-height			ANOVA^a^		
	**Child morbidity at 3 and 6 months**													
		Diarrhea	Nominal			Categorical			% of children with diarrhea			Risk ratio		
		Fever	Nominal			Categorical			% of children with fever			Risk ratio		
		Cough	Nominal			Categorical			% of children with cough			Risk ratio		

^a^ANOVA: analysis of variance.

### Data Collection Tools and Procedures

#### Data Collection Plan

Application of baseline tools and procedures will be repeated by the field staff who participated in baseline data collection to collect data from both arms. Before the data collection, a 1-day refresher training session will be organized for the 8 data collectors and 2 field supervisors. Recruitment of field staff was on the basis of proficiency in French, English, and Fulfulde (the dialect of the Mbororo people), familiarity with the Mbororo culture, and prior experience with surveys.

#### Caregivers’ Interviews

Face-to-face interviews will be conducted by trained data collectors (interviewers) using pretested interviewer-administered questionnaires. The interviewer-administered questionnaires were adapted from the UNICEF (The United Nations Children's Fund) multiple indicator cluster surveys tool [18]. The adapted questionnaire was prepared in English and then translated and back-translated into French and Fulfulde. The components of the questionnaire are household and caregiver’s sociodemographic characteristics, child demographic and health status characteristics, complementary feeding practices, and WASH facility and practices. Based on the health belief model constructs, the questionnaire contains 4 questions on caregiver’s perception of child susceptibility to malnutrition, 4 questions on caregiver’s perception of the severity of child malnutrition, 6 questions on caregiver’s perception about barriers to practicing appropriate complementary feeding and WASH, 4 questions on caregiver’s perceived benefits of appropriate complementary feeding and WASH practices, and 4 questions on caregiver’s self-efficacy to implement recommended actions. Each interview is estimated to last between 50 to 60 minutes and will be conducted according to the time, day, venue, and language convenient to the interviewee. The interviewers will read out the questions to the respondents and complete the questionnaires accordingly.

#### Anthropometric Measurements

Pretested portable anthropometry tools (battery-powered digital infant and toddler weighing scales, stadiometers, measuring tape, and lying wooden boards) will be used. The weight and height/length measurements will be performed using standard procedures [19]. Lying or sitting weights for children aged 0-23 months will be measured to the nearest 0.01 kg, and standing weights for older children will be measured to the nearest 0.1 kg. The weighing scale will be calibrated to 0 before each measurement. Recumbent lengths for children aged 0-23 months will be measured to the nearest 0.1 cm with measuring tape and lying boards placed on a flat ground surface. Standing heights for older children will be measured to the nearest 0.1 cm, with the head, shoulder, buttock, and heel touching the vertical surface of the stadiometer. Measurements will be taken twice and the mean recorded.

### Data Analysis Plan

Like the procedure at baseline, 1 biostatistician and 2 trained data entry clerks will be responsible for the endline data management and analysis. Data will be cross-checked for inconsistencies (outliers and extreme points) and errors and incompleteness before, during, and after manual entry into SPSS version 23 (IBM Corp). The data will be further treated for reverse coding, potential points, and outliers, as well as missing data, before being exported to SmartPLS2 for further investigation and fitting of the structural model.

Sociodemographic and child morbidity data will be analyzed and summarized using descriptive statistics. The data will be expressed as mean (SD) or median (range) for continuous variables and as a number (percentages) for categorical variables. The *t* test and analysis of variance for comparing group means will be used for primary and secondary outcome variables. A chi-square test will be applied to analyze the categorized variables. Child nutrition outcomes will be computed from anthropometric indices using Stata version 11 (StataCorp) and compared with the WHO 2006 growth standard median [20].

Results for group comparisons will be expressed as a risk ratio for binary outcomes, corresponding to 2-sided 95% CIs, and associated *P* values. *P* values will be adjusted to 2 decimal places with values less than .01 reported as <.01. Adjusted analyses using baseline variables will be performed using multivariate logistic regression to determine the continuing influence of key baseline characteristics on the outcomes. The Kaplan-Meier survival analysis will be used for timed variables like morbidity. Intention-to-treat analysis will be used, and the clustering effect will be considered in the analysis. All analyses will be performed at a 95% CI. The significance level will be set at a *P* value <.05.

### Ethical Considerations

Ethical approval (reference no. 2019/1002-07/UB/SG/IRB/FHS) for the study was obtained from the Faculty of Health Sciences Institutional Review Board at the University of Buea. Administrative authorization was sought from the West Regional Delegation of Public Health. Informed verbal and signed consent and parental assent will be obtained from study participants before inclusion in the study. Participation will be voluntary with participants being able to withdraw from the study at any time. Anonymity and confidentiality will be assured and maintained.

### Dissemination Plan

The results will be disseminated to internal and international audiences through publications in peer review journals, open access publications, and national and international conferences. Compensation will be provided in the form of workshops for those communities participating in the study.

## Results

From the preintervention study undertaken from August 2019 to September 2019, baseline data were collected and partially analyzed to inform the design and implementation of the intervention study. In February 2020, 10 Mbororo communities with 242 child–caregiver pairs were selected for the trial, while 6 CNVs and 6 CHWs were selected and trained to lead the trial. Data collection and analysis are ongoing, and results are not available for this manuscript.

## Discussion

Nu-WASH is a multicomponent health education model that will be delivered simultaneously to caregivers in the experimental arm to improve their nutrition capacity and child nutrition outcomes. To enhance its acceptability, sustainability, and cost-effectiveness, the intervention is culturally tailored for delivery in the community setting through CPGs, and led by trained female Mbororo CNVs and existing CHWs. The language of communication is predominantly Fulfulde. Additionally, dietary diversity for young children will be based on locally available and consumed food items in various communities. It is expected that caregivers will perceive child undernutrition as an important health concern and will be motivated to practice recommended actions to bring about a positive behavioral change.

Theory-based behavioral change communication to promote healthy feeding practices is central to interventions that are aimed at improving infant and young child nutrition [8]. Our education intervention is based on the health belief model. The model posits that people will take action to prevent a negative health outcome if they regard themselves as susceptible to a condition, if they perceive a negative health outcome to be severe, and if they perceive that the benefits of adopting a particular behavior outweigh the perceived barriers they need to overcome [21]. The chances are higher if they are exposed to factors that prompt action (cues to action) and if they have the confidence to take action (self-efficacy) [21]. Based on these constructs, the intervention module is designed to enable participants to perceive malnutrition as a severe yet preventable condition that can affect their children (susceptibility). Additionally, skills acquisition will enhance their self-efficacy to overcome some perceived barriers and to take recommended action, leading to a positive behavior change. We predict that our community-led and culturally sensitive Nu-WASH intervention will improve caregivers’ complementary feeding KAP and child nutritional outcomes as compared to routine health facility–based nutrition education among the study population.

## Abbreviations

CHW: community health worker
CNV: community nutrition volunteer
CPG: caregivers’ peer support group
KAP: knowledge, attitude, and practice
Nu-WASH: nutrition, water, sanitation, and hygiene
WASH: water, sanitation, and hygiene
WHO: World Health Organization
UNICEF: The United Nations Children's Fund
